# Sketch of the Medical Topography of Malta

**Published:** 1825-03

**Authors:** 

**Affiliations:** Deputy Inspector of Hospitals; Author of the "History of the Plague of Malta," &c.


ART. VIII.—*Sketch of the Medical Topography of Malta.* By —— TULLY, Esq. Deputy Inspector of Hospitals; Author of the "History of the Plague of Malta," &c.

[Continued from vol. lii. page 481.]

To me, then, it appears that the causes which annually produce fever at Puales, are so strikingly marked, as to be distinctly divided into exciting and predisposing: the first from causes not generally understood, and the latter from the constant exposure of the peasantry to night-watching, and to the sun's rays, without adequate nourishment. These causes we shall now endeavour to explain. In the first instance, on visiting this ground, our attention is attracted by the drains we have noticed as intersecting the little uncultivated space; and these drains have evidently been made with great attention, as we trace each of them to distinct openings of masonry in the embankment, intended to afford free exit to the water drained from the neighbouring ground: but these mouths, or outlets, have been filled up, from time to time, by sea-weed and rubbish thrown up during the north-east winds, to which the extremity of this harbour, like that of La Valette, is exposed. In consequence, therefore, of these interruptions, which have been gradually increasing for years, these drains have become luxuriant in all sorts of vegetable productions, issuing from a miry bed. Yet these drains, crammed even as we found them, and acted upon as they must be by the summer heat, are by no means equivalent to the production of the annual fevers of Puales; as the vegetable effluvia arising from these cuts could not, from the very small extent of surface exposed, extend its influence beyond their own lines. Besides, the residences of the people are a good deal removed; and it is not very likely that the peasantry, who pass their nights in the grounds, as guards over their produce, and the persons chiefly affected by the remittent fever, would select the immediate vicinity of these drains as their stations, where there is nothing whatever to be guarded.

A much greater source of evil than these cuts, appears to me to exist in the large bank of sea-weed thrown up at the extremity of these grounds, offering both a depth and extent of surface for the exhalation of a far more deleterious effluvia than could possibly be exhaled from the whole of the cuts, even were their surfaces united. Besides, a quantity of this sea-weed, frequently in a state of great moisture, is deposited upon the neighbouring soil, to serve as manure. But I do not even consider that the combined effects of these causes are at all sufficient to explain to us how these fevers are produced: but, pursuing our investigation, we shall find that the unusual sickness in this neighbourhood owes its origin to others than the causes enumerated.*

We have already shown the peculiarities of the lands of Puales, from which it must be evident that, at all seasons of the year, they must be subject to an excess of moisture,—not from any lodgments of water, but from the succession of streams issuing from the various springs. These streams, collecting as the water proceeds to its level, passes over not only the uncultivated part, but a large portion of the cultivated grounds, by which much is drank up, from the aptitude of the soil: now, the constant evaporation and exhalation that must necessarily follow during the summer heats, cannot, in my mind, fail to produce, under certain circumstances, a disease similar to that arising from marsh; and these certain circumstances I consider to be the daily exposure of the peasantry to the full influence of heat,—to the exposure of the night-dews, sleeping upon the moist ground,—and to a degree of exhaustion towards night, arising from the constant fatigue both of labour and watching; to which we must not omit adding, a singularly poor, nay wretched, food, consisting in summer, chiefly of water-melons, cucumbers, and black barley-bread.

That these causes of fever are much more general than is usually understood, there is much reason for believing; for we frequently find these fevers assailing us in spots where nothing like marsh can be traced. Thus, the most elevated situations, and best cultivated tracts, have been known, at different periods, to produce, in warm climates, the most fatal fevers; but in all these instances, by reflecting upon what has been just said, we may be enabled to trace the cause which produces disease in both situations. In the higher grounds, I have discovered that the

* That neither of these causes contribute to produce the remittent of Puales, may be inferred from the exception of the Maltese soldiers, stationed in the immediate vicinity, from fever.

moisture retained by the olive-trees, growing crowded together upon a rich soil, was sufficient to produce those exhalations that became so dangerous to the inhabitants immediately upon the spot; and I have observed a neatly-arranged garden, well watered, produce fever, in the autumnal months, in the ill-fed labourer, whilst the lord and pampered menial totally escaped, —and upon what principles I shall now endeavour to explain.

The history of diseases in all parts of the world, with which we are acquainted, confirms the fact, that the highest habitable mountains frequently, without the presence of any epidemic, give rise to fevers of a similar type with those of the marshy plain; and we have many proofs that the town-girthed beach will, under circumstances, expose those living in the immediate vicinity to similar danger. In explanation of what is here advanced, I shall refer to Clonio, in Corfu, the highest habitable point in the island, and the little beach barracks adjoining the mole of Zecute, both places notoriously unhealthy at particular periods during the summer months, but more especially Clonio, where remittent fever annually commits its ravages; and, indeed, of the latter I may observe, that so strongly was the unhealthiness of that quarter impressed upon my mind, that, when surgeon of the 35th regiment, many years since, I recommended the removal of the regiment from the Mole barracks, and another in the same line, in consequence of the appearance of fever amongst the men at those stations: fortunately, my suggestions were immediately attended to, and disease consequently disappeared. Now, in neither of these places are there any vestiges of marsh to be discovered. Certainly, the eye may reach the swampy valley of Ropo, and sometimes at night the brilliant and busy fire-fly skipping above its surface, beneath the lofty and pinnacled cloud; but this source of danger is so far removed, that we cannot well imagine that any connexion between these places can exist, connected either with health or disease; for, if we admit any such connexion, we must, in my mind, first reverse the law by which we hold the exhalations from the earth to be governed,—a position which I am inclined to believe will not be readily granted; for, in doing so, we must ascribe to these exhalations a much more aërial ascent than is consistent with our general reasoning upon this subject. But, even admitting that such was possible, the admixture of these exhalations with pure air, in distances so removed from the surface, as we may say, of the diseased earth, would most likely render these exhalations innocuous: therefore we cannot, with any appearance of truth, attribute any such peculiarities to marsh miasma: indeed, it appears to me that there are many circumstances which render this altogether impossible; for we have strong reasons for concluding that the exhalations from the earth in marshy soils in hot countries during the day, descend with the evening dew. That this is the case, is made probable from the dense, fog-like vapour which begins to hover over swamps, when the influence of the sun is on the wane, and which continues buoyant over the surface of the earth until the sun again begins to exert its influence: and, in support of this assertion, it is only necessary to refer to the well-known fact of the frequent exemptions of persons inhabiting the upper apartments of houses, in marshy situations, from the diseases incidental to marsh, whilst the ground tenants shall not escape.

If, then, what is here stated be admitted, we shall at once discover why the effluvia arising from moist grounds in warm climates, at certain seasons of the year, particularly when aided by the predisposing causes we have already mentioned, becomes not only capable of exciting, but peculiarly fertile in producing, disease; and why the influence of this effluvia is more particularly exerted upon those exposed to its action during the night; and at once unfold to us the cause of disease in various situations apparently healthy, which would otherwise appear inexplicable.

The effluvia from these causes may require a longer exposure to their effects, to produce a derangement of the system, than the miasmata of marsh; but, although the progress may be slow, morbid action will scarcely fail to follow, when the predisposing causes are so powerful as we meet with in general in these countries,—unless, indeed, some remedy be applied sufficient to remove the most dangerous of these predisposing causes.

To the soldier, this observation is more applicable than to the poor peasant, whose mode of life is scarcely expected to be varied,—at least in a degree sufficient to protect him from the danger to which he is so much exposed; but when the soldier becomes exposed to danger, similar to what has been now noticed, and which unfortunately recently occurred in the island of Zante, a remedy might perhaps be applied; such as preventing the whole of the troops occupying such situations, with the exception of those on duty, from being out of their quarters after sun-set; prohibiting all those on guard, or out-lying picquets, from sleeping without their guard-room, or in the open air; furnishing these guards with segars, which even the sentries should not only be permitted, but recommended, to use; and the issue of a small allowance of spirits to each man, on his being posted as sentry, an arrangement which might be so regulated that no abuse whatever could follow. Precautions like these appear to me to merit attention, as a means of preventing disease in exposed situations during the autumnal nights, and probably of saving many lives. These precautions would, of course, be considerably aided by opposing every possible obstacle to drunkenness, particularly during the summer months; thereby removing one of the most powerful predisposing causes to disease in the soldier.

In concluding this part of our subject, it is presumed that it has been satisfactorily proved that the causes which have at former periods produced disease amongst the inhabitants residing in the vicinity of the Marsa, have arisen from the rapid decomposition of the marine matter with which that ground had for a long series of years been amply supplied, and frequently renewed, producing both remittent and intermittent fevers during the seasons when these diseases prevail; and that the remittents of Puales are totally unconnected with marsh miasma, but that they owe their origin to the operations of those combined causes which we have just enumerated; and, further, that those wandering cases of remittent fever, that we are told are occasionally to be met with in other parts of the island, of which I have no knowledge, if fairly examined, will be found to be dependent upon the same causes: unless, indeed, they are traced to visceral obstructions, so prevalent amongst an ill-fed peasantry, and so common a source of the autumnal bilious remittent.

In the progress of this sketch, the climate has been necessarily noticed, but in so very cursory a manner, that I feel the subject to be one of too much importance to be passed over without some further observation. Pursuing, then, this intention, we find, upon examining the history of Malta, that the climate, from the earliest period, has been considered by each succeeding writer as particularly favourable to the health of its inhabitants, and as by no means uncongenial to the foreigners. Ciactar, in his History of the Island, states the ordinary period of life of the inhabitants to be from eighty to ninety years; and Pecacchi, a very ancient author, speaking of the inhabitants of Malta, expresses himself in the following terms:—"Plerosq; senectus octoginta annorum, non morbus dissoluit." In later years, British experience has contributed much to our general information respecting the island, and the nature of the climate has not escaped the attention of our professional brethren, who have occasionally resided here; and, from their concurrent and favourable testimony, it may, with much propriety, be inferred that they have truth upon their side. Indeed, I may, with much safety, venture to affirm that, in our medical research, we shall have as little cause to complain of the climate as any other part of our subject; although, in doing so, I am fully aware that I am warring against fashionable opinion, which is decidedly hostile to the climate of Malta. This belief, however, I am inclined to attribute more to the indolence and ennui attendant upon a small insular residence amongst this class of society, than to any thing like reality. In saying thus much, I do not mean to be understood as considering Malta, in point of climate, in every respect unexceptionable; for this would be ascribing a degree of perfection to it, which few parts of our habitable globe possess; but that the climate is favourable, not only to the inhabitants, but to the foreigner who arrives here with an unbroken constitution.

To the hypochondriac, as far as his own feelings are concerned, every climate may be said to be alike; and, generally speaking, we may apply the same observation to the enervated and diseased frame; as those unfortunately labouring under either of these states, for the most part, view nature, even in her fairest form, through a distorted medium, and look upon the climate where they experience their sufferings as far worse than all others. It is not, therefore, to the opinions of such as I have here described, (which, I fear, is too frequently the case,) that we are to bend in forming our decisions upon so important a point; but rest them upon less exceptionable sources, aided by our own experience.

M. Dolornieu, in his Essay upon the Temperature of Malta, leads us to infer that the summer heats of the island are peculiarly distressing; and he is the only modern author, with whose writings I am acquainted, who states that both natives and foreigners alike feel its effects. Although I coincide with him entirely as to the oppressive sensations produced by the summer heats upon foreigners, yet I can by no means agree with him in his assertions in regard to the natives; for, not only their being assimilated to the climate, but also their mode of living, renders them far less exposed to the influence of these periodical heats than the unseasoned occupant, and certainly in no comparison less so than the inhabitants of our own latitudes. For my own part, I consider that we may, with some degree of propriety, compare the inhabitants of particular countries to their indigenous produce: remove them from their native soil, they flourish for a time, and then degenerate. This observation is daily exemplified in many of the products of our own country, transplanted to this island, where they generally thrive for the first year; the second, they prove far inferior; and for the most part, after the third year, they are unfit for use. Man, in my opinion, will alike, sooner or later, feel the influence of the change upon his constitution; but this cannot, with any justice, be attributed to the defect of climate, but to a want of assimilation, unless it can be satisfactorily proved that a defective state absolutely exists.

Now, although I have concurred with the general opinion of the oppressive sensations complained of by most persons unaccustomed to the climate, during the summer months,—or rather let us say, from the middle or latter end of July to the end of September,—and noticed, as respects strangers, by M. Dolornieu; nevertheless we find that these sensations are quite at variance with the actual temperature of the air, as indicated by the thermometer; for, upon examining the registers kept with much attention by various individuals, residing both in town and country, with those of our own department, it appears, from the average of the last five years, that the ordinary range of the thermometer is from eighty to eighty-three degrees of Fahrenheit (though there are instances of the mercury rising as high as 106°,) during the summer; and the standard during the winter is about fifty-two degrees; but I have known it for some days as low as forty-five, with a keen and cutting north-west wind. Yet it frequently occurs, within both these periods, that our feelings would lead us to conclude the thermometer to be very many degrees beyond the actual standard.

The winds which prove the most distressing in Malta, are the sirocco, or south-eastern, and the south winds; and, when they blow during the summer months, they are generally steady, with little variation in the heat. The former are accompanied with a damp and heavy atmosphere, and the latter with a dry and parched sensation; both extremely relaxing, and particularly felt by the invalid: more so, as they are not unfrequently followed, and as frequently preceded, by a light north-west breeze, with a sudden variation in the temperature of fifteen or twenty degrees. There is a more popular dread of the south-east wind, or sirocco, during the summer months, than of the south winds; but this is solely to be attributed to its duration, for certainly, and in point of fact, the south winds are by far the most distressing; but fortunately they seldom continue to blow more than three days, and then sometimes only between sunrise and sunset, when a refreshing sea-breeze from the northward sets in, dying away towards morning. The great difference experienced in Malta between these winds, may be accounted for from our proximity to the continent of Africa, from whence we have the south winds, passing over a burning desert, reaching us unrefreshed, from the short distance it has to traverse: and, when it does reach us at the opposite face of the island, it comes to us parched anew, from the heated soil it has to pass ere it approaches the capital, or other inhabited parts of the island; wherefore, this wind must contain much less humidity than the sirocco. The effects, however, of both winds upon the human frame, from the cause just mentioned, we find to be pretty much the same, as noticed both in hypochondriacs and those labouring under chronic visceral affections, particularly remarkable in the attendant great despondency, with an increase of the more urgent symptoms; owing, as we presume, to certain atmospherical causes, producing increased action of the heart and arteries, and derangement of the perspiratory organs; and these alterations continue until the cause is removed. To these changes, and the high range of the thermometer during the summer months, may be attributed those marked effects which are produced, during this season, upon those labouring under pulmonic and hepatic affections, and which renders Malta (as experience has so fully proved) decidedly hostile to the residence of persons thus unhappily situated.

It must be understood that, although the climate of Malta is subject to great and sudden changes, yet that these transitions are by no means frequent. I make this observation purely from my own knowledge, resting upon nearly a four-years' residence, and from an accurate examination of records of the weather. It is not, therefore, to be expected, that much evil can arise from the vicissitudes experienced in this island. The inhabitants, during these changes, apprehend only what they denominate a *colpo di vento*, which they endeavour to guard against by a cautious attention to clothing: and certainly they ought to be but little dreaded by those who are accustomed to the very rapid and considerable diurnal atmospherical vicissitudes of our own climate.

The winds here noticed do not confine their influence alone to the invalid or valetudinarian, but extend also to the vegetable kingdom: the agriculturists dread their effect upon their crops, for, when they blow for any continuation, especially the sirocco, a blight is frequently the consequence; both corn and cotton alike suffering,—the former between the months of May and June, and the latter from August to September. Further, the south wind is peculiar in its action upon the native rock, as we observe the stones forming the ancient buildings and works, exposed to its action, in many parts in a state of decomposition, demonstrated by the honey-combed appearances of considerable portions of their respective surfaces; whilst the other faces of these buildings and works do not exhibit it, at least by no means to the same extent as is witnessed in these particular exposures.

Of these winds still more might be said: as the effects of the sirocco upon meat, disposing it to run rapidly into a state of putrefaction, &c.; also upon wine, which frequently turns sour if bottled during the prevalence of a south-eastern, as in some instances in thunder-storms: and it has been even asserted that it possesses the property of preventing salt from crystallising. But there are few climates that do not possess some peculiarity, and few with the advantage of these peculiarities not militating against health, which there is much reason for believing to be the case in Malta. In England, for example, the east wind is known to exert a considerable influence on the human frame, both mental and corporeal; and Dr. Johnson, in his work on the Influence of the Atmosphere, says that there is something in an easterly wind, independent of its temperature, which is inimical to the free and regular performance of the animal functions, and particularly the functions of the skin; and that invalids will feel its effects in rooms, where the temperature is regulated by a thermometer, nearly as much as if they were in the open air. However the invalid may suffer from the effect of our siroccos and southerly winds, our experience does not offer any proof of its mental or corporeal effects upon the sound constitution; nor is there, as far as I am acquainted, any evidence of the fatal consequences of those effects, in acts of suicide among the natives.

Whatever may be the impurities with which our obnoxious winds are charged, we have the satisfaction to know that their effects are by no means of any duration, and they leave behind them very little impressions beyond the momentary feelings they produce; and that, in their course over our little territory, they imbibe nothing from the soil that can prove in the least inimical to health: to which we can add another very important consideration, that of the gradual change of seasons, which in general is such as to allow the constitution to adapt itself to the approaching alteration. This, of course, like every other rule, is liable to exceptions; but, generally speaking, we shall find the observation to be correct, and what is supported by the ordinary custom of the garrison, as we find that, on the approach of winter, it is invariably ordered that troops on guard should wear their cloth trowsers between sunset and sunrise; the troops off duty require no such change: and this system is generally pursued from a month to six weeks, until the change of season is fully established, when the winter clothing is put in wear. Indeed, our seasons run their course with as little alteration as we meet with in the healthiest climates. That some irregularities will occasionally occur, cannot be questioned; but, as far I have observed, they never have been of a character to influence health.

The rainy seasons, unlike those in the Ionian Islands, are unattended with danger; they neither occasion sickness by partial falls, nor remove it by their continuance: they come and pass off without occasioning any other feeling than the temporary inconvenience. Rain, as far as I have observed, generally sets in about September, and continues there but for a few days. Occasional heavy showers mark the intervening period until December, when again heavy rains occur; and from this period until the end of March, or sometimes middle of April, we are not free from wet: yet there is no constancy in the rains, and it frequently happens, throughout a winter, that the greatest quantity of rain falls during the night. Contrary to most other places, the north-east appears here to be the wet quarter; falls of rain generally accompanying the heavy gales from that point, known to us by the name of *grecale*, and dreaded by the mariners, from the havoc it commits even within the ports.

Thunder and lightning are by no means so frequent as in the neighbouring islands and continents: nor is this to be wondered at, when we consider the nature of the little spot we occupy, without a single point of land high enough to attract a passing cloud. The atmosphere, however, is generally under the influence of these revolutions about the equinoctial changes; but the effects are seldom productive of any serious mischief: the worst I have known having been confined to partial injuries to shipping in the harbour, and even this rather a rare occurrence.

The dews are extremely heavy in Malta, and appear to supply, at particular seasons, the want of rain. They are more remarkable at those periods when the soil requires moisture, as in spring and the latter end of autumn; and I do not know how true the observation may be, but I have frequently remarked that, at these seasons, the dew was far greater during the middle quarters of the moon, than at any other period: perhaps, this may have some connexion with the process of vegetation. The dews of summer are by no means so heavy as might be supposed, and certainly by no means equal to what I have noticed in other countries, in similar latitudes. That this is the case, may be inferred from the custom of sleeping in the open air, so very prevalent during the summer nights in Malta: and it is one of the most convincing proofs we can adduce, both of the dryness of the atmosphere and the non-existence of unhealthy exhalations; for it is a positive fact that no precautions whatever are taken in Malta against the night-air during summer, many of the higher ranks remaining abroad, and in their balconies, till after midnight, whilst thousands of the middling and lower classes are known to sleep upon their terraces and outside their doors, both in town and country: and all this at the very time when it is undeniably proved that, where causes of insalubrity exist, they are not only abroad, but acting with their greatest vigour; and surely it is but fair to conclude that this prevailing custom of nocturnal exposure must be, at this particular season, entirely free from danger, otherwise it would not be so very generally sanctioned as we find it is.

The nature of the ordinary diseases of the inhabitants also proves the nature of the climate: they are mostly fevers of the continued form, diarrhœa, dysentery, and pleurisy, seldom running to fatal terminations; which is the more to be wondered at when we reflect upon the very inert practice of the native physicians. Of the remittents and intermittents I have already spoken; yet I find I have omitted one of the principal causes of the appearance of those diseases upon the face of the Civil Hospital Returns,—namely, the introduction of those diseases by the boats' crews employed in commerce with the coast of Sicily; a more frequent occurrence than we are aware of, and to an extent beyond general belief. Of the truth of this statement there can be no doubt, as it rests upon the authority of a British officer employed in the principal duties of the civil hospital, who has assured me that, within the last two months, instances have occurred where the whole of several boats' crews were attacked with remittent and intermittent fever, on their retnrn from the coast of Sicily, attested by their previous quarantine, and subsequent admission into hospital.

[To be continued.]

